# Cannabis use in a Canadian long-term care facility: a case study

**DOI:** 10.1186/s12877-024-05074-2

**Published:** 2024-05-29

**Authors:** Lynda G. Balneaves, Abeer A. Alraja, Genevieve Thompson, Jamie L. Penner, Philip St. John, Daniella Scerbo, Joanne van Dyck

**Affiliations:** 1 College of Nursing, University of Manitoba, Winnipeg, MB Canada; 2 Max Rady College of Medicine, University of Manitoba, Winnipeg, MB Canada; 3 Riverview Health Centre, Winnipeg, MB Canada; 4 College of Nursing, Rady Faculty of Health Sciences, University of Manitoba, 89 Curry Place, Winnipeg, MB R3T 2N2 Canada

**Keywords:** Medical cannabis, Non-medical cannabis, Long-term care, Residential care, Older adults

## Abstract

**Background:**

Following the legalization of cannabis in Canada in 2018, people aged 65 + years reported a significant increase in cannabis consumption. Despite limited research with older adults regarding the therapeutic benefits of cannabis, there is increasing interest and use among this population, particularly for those who have chronic illnesses or are at end of life. Long-term Care (LTC) facilities are required to reflect on their care and policies related to the use of cannabis, and how to address residents’ cannabis use within what they consider to be their home.

**Methods:**

Using an exploratory case study design, this study aimed to understand how one LTC facility in western Canada addressed the major policy shift related to medical and non-medical cannabis. The case study, conducted November 2021 to August 2022, included an environmental scan of existing policies and procedures related to cannabis use at the LTC facility, a quantitative survey of Healthcare Providers’ (HCP) knowledge, attitudes, and practices related to cannabis, and qualitative interviews with HCPs and administrators. Quantitative survey data were analyzed using descriptive statistics and content analysis was used to analyze the qualitative data.

**Results:**

A total of 71 HCPs completed the survey and 12 HCPs, including those who functioned as administrators, participated in the interview. The largest knowledge gaps were related to dosing and creating effective treatment plans for residents using cannabis. About half of HCPs reported providing care in the past month to a resident who was taking medical cannabis (54.9%) and a quarter (25.4%) to a resident that was taking non-medical cannabis. The majority of respondents (81.7%) reported that lack of knowledge, education or information about medical cannabis were barriers to medical cannabis use in LTC. From the qualitative data, we identified four key findings regarding HCPs’ attitudes, cannabis access and use, barriers to cannabis use, and non-medical cannabis use.

**Conclusions:**

With the legalization of medical and non-medical cannabis in jurisdictions around the world, LTC facilities will be obligated to develop policies, procedures and healthcare services that are able to accommodate residents’ use of cannabis in a respectful and evidence-informed manner.

**Supplementary Information:**

The online version contains supplementary material available at 10.1186/s12877-024-05074-2.

## Background

In October 2018, Canada became the second country to legalize non-medical cannabis [1]. Despite the increasing interest in cannabis among Canadians of all ages [2], the percentage of individuals over the age of 15 years reporting cannabis use a year following legalization remained relatively unchanged at 18% [3]. The only age group to report a significant increase in cannabis consumption was those aged 65 + years, with 7.6% reporting cannabis use in the past 3 months [3] in 2019 compared to 4% in 2018. This upward trend in cannabis use among Canadians 65 years or older was also observed in 2021 [4].

This increase may reflect a growing acceptance of cannabis among older populations who were previously dissuaded from taking cannabis due to its illegal status as well as limited accessibility through legal means. In addition, the rise in cannabis use among older adults may reflect a harm reduction approach, substituting cannabis for other recreational substances with substantial health risks, such as alcohol [5]. Moreover, the belief in the potential therapeutic benefits of cannabis [6–8], such as the management of pain and sleep issues, is becoming increasingly prevalent among older adults. There has been limited research, however, among older adults in Canada to understand this progressive trend in cannabis use and the influencing factors [9].

Canada has been a world leader in cannabis legalization, launching a federal medical cannabis program in 2001. Since this time, the medical cannabis program has undergone numerous revisions, including how authorization is obtained, what types of products are available, and where cannabis is purchased. Currently, Canadians can seek medical authorization from either a physician or a nurse practitioner, and access a variety of cannabis products, including dried flower, capsules, and oils, which are purchased online through a licensed producer (LP). Some individuals also apply for a personal or designated grow license to produce their own supply of dried cannabis. Outside of the medical authorization program, individuals can access non-medical cannabis through an authorized storefront. It is estimated that over 1 million Canadians are using cannabis for therapeutic purposes [4], with 247,548 individuals officially registered as of March 2022 [10]. Among the 479,400 individuals over the age of 65 who reported cannabis use in the third quarter of 2019, 52% utilized cannabis exclusively for medical reasons, and another 24% reported using cannabis for both recreational and medical purposes [3].

Despite the growing interest in cannabis as a therapeutic agent, there has been limited human research due to its illegal status in many countries, as well as the challenges posed by the complexity of the cannabis plant compared to single agent, pharmaceutical forms of cannabis (e.g., nabilone) [11, 12]. Notwithstanding these challenges, there is emergent research on the potential role of cannabis-based medicines in the management of health conditions common among older adults, including osteoarthritis [13], sleep disorders [14], dementia [15], and Parkinson’s [16, 17], which are also prevalent among individuals residing at long-term care (LTC) facilities. For example, several studies have found cannabis-based medicines to significantly reduce neuropsychiatric symptoms and improve quality of life among people living with Alzheimer’s Disease [18–20]. Cannabis may also play a significant role at end of life in not only alleviating physical symptoms, such as pain, nausea and vomiting, and appetite loss, but also addressing the emotional and existential issues that may arise [21]. It has also been proposed that cannabis may have a therapeutic role among rehabilitative populations who often reside in LTC settings, including those with spinal cord injuries [22, 23] and traumatic brain injury [24]. The evidence base surrounding cannabis as a therapeutic agent, however, remains limited with few large randomized clinical trials conducted to date.

Cannabis is not a benign substance and may pose risk to older adults, especially those living with frailty or cognitive impairment. Given the known cognitive effects of tetrahydrocannabinol (THC), a cannabinoid found in many forms of cannabis, adults living in long-term and rehabilitative care settings may experience somnolence, confusion, and fatigue [25]. Cannabis high in THC may also negatively impact motor coordination and increase the risk of falls, especially among those with impaired balance and walking ability [25]. As research advances on cannabis, there has been growing awareness of its negative interactions with certain medications [26], which can pose a significant issue among older clients prone to polypharmacy. Lastly, numerous health conditions are contraindicated with cannabis use, including heart disease, and a personal or family history of psychosis, schizophrenia, or bipolar disorder [27].

Despite limited research with older adults regarding the therapeutic benefits of cannabis, there is increasing interest and use among this population, particularly for those who have chronic illnesses. As adults age, they are more likely to experience multimorbidity, and a significant number of older adults spend their last years of life residing in a LTC facility [28, 29]. LTC facilities are, thus, placed in a unique position. While these facilities are considered medical institutions that provide evidence-informed supportive health care, they have also become home for individuals who are no longer able to reside safely in the community. Increasingly, these types of facilities are challenged to create home-like environments and offer residents the opportunity and autonomy to engage in potentially risky health behaviours [30]; behaviours that individuals in the community have the independence and legal right to choose, such as alcohol or tobacco consumption. With the legalization of non-medical cannabis and the growing interest in the potential of cannabis to manage challenging health conditions, it behooves LTC facilities to reflect on their care and policies related to the use of legal substances, such as cannabis, and how to address residents’ cannabis use within what they consider to be their home.

The overarching aim of this case study was to understand how one LTC facility, and its healthcare professionals (HCPs) and administrators, addressed the major policy shift in Canada related to medical and non-medical cannabis. Specific research questions included: (1) What are the experiences and perceptions of HCPs and administrators regarding the use of medical and non-medical cannabis at LTC settings?; (2) What are the perceived barriers/facilitators to medical and non-medical cannabis use at LTC facilities from the perspective of HCPs and administrators?; and (3) What are the educational needs, attitudes, and practices of HCPs at LTC facilities related to medical and non-medical cannabis?

## Methods

### Research design and setting

An exploratory case study design was utilized in this study. This type of case study is used to explore those situations in which the phenomenon being evaluated has no clear or single set of outcomes [31]. The case selected for this study was a large LTC facility in Western Canada. This 387-bed residential facility provides 24/7 care to a diverse population, including older adults with cognitive and physical disabilities, individuals recovering from stroke and traumatic brain injury, and those requiring end-of-life care. Individuals with these various conditions may reside in several units, including palliative care, rehabilitation, personal care home, and complex chronic care. The case study included an environmental scan of existing policies and procedures related to medical and non-medical cannabis use at the LTC facility, a quantitative survey of HCPs’ knowledge, attitudes, and practices related to medical and non-medical cannabis, and qualitative interviews with HCPs and administrators. The qualitative interviews were informed by qualitative descriptive methodology [32] and explored HCPs’ and administrators’ experiences, beliefs, perceptions regarding cannabis use in LTC, and the related barriers and facilitators.

### Sample and recruitment

For the survey, a convenience sample was drawn from the entire population of accredited HCPs working in the selected facility. Eligibility criteria included being 18 + years, able to read/speak English, currently employed and providing care at the LTC facility, and able to provide informed written consent. Study participants were recruited through an emailed letter of invitation, posters placed in staff areas, and in-person presentations by a research assistant. From participants who took part in the survey, a subsample of HCPs, including administrators, who expressed interest in taking part in an interview was selected. The data collection period was from November 2021 and August 2022.

### Data collection

For the environmental scan, facility administrators were approached via an emailed letter and asked to identify relevant policies and procedures related to cannabis use within their LTC facility. Policies relevant to both residents’ use of cannabis and HCPs’ practice related to medical and non-medical cannabis were requested. Provincial and federal cannabis policies were also collected.

The survey was modified from a questionnaire utilized in two national studies that examined Canadian physicians’ and nurse practitioners’ knowledge, attitudes, and perceptions of the associated barriers and facilitators related to medical cannabis use, as well as their preferences regarding medical cannabis education [33, 34]. This survey has been found to be internally consistent, with Cronbach’s alphas of 0.70 to 0.92 reported across subscales [33, 34]. Slight word changes were made to reflect the fact that individuals living in LTC facility are referred to as residents, not patients, and the name of the facility was used to orientate the questions towards HCPs’ attitudes and practices related to cannabis use within the LTC setting.

Survey items were added that assessed HCPs’ practices related to addressing residents’ and family members’ questions about cannabis, as well as requests for medical cannabis authorization and follow-up care. A demographic survey that assessed gender, age, professional designation, years in practice, area(s) of practice, and education related to medical cannabis was included. The survey was available in hard copy (Supplementary Material 1) as well as online through the software program, Qualtrics®.

An interview guide was developed by the research team, which included a facility administrator and HCP, and was informed by the literature and previous cannabis research conducted by members of the research team [35] (Supplementary Material 2). Due to the COVID-19 pandemic, all but one interview was conducted by the project coordinator (AAA) via Zoom, with one interview occurring over the phone. The interviews were 20–30 min in length and were digitally recorded and transcribed verbatim. Both the survey and interview were completed at times preferred by the respondents, including within and outside work time. No honoraria were provided for study participants.

### Data analysis

The policies identified through the environmental scan were reviewed and summarized in table format, with similarities, contradictions and gaps identified.

Quantitative survey data was uploaded into the statistical program, SPSS® v.25. Descriptive statistics were used to summarize demographic information, knowledge about medical cannabis and related attitudes, perceived barriers and facilitators, practice experiences, and preferred educational approaches.

Perceived knowledge gap was calculated by computing the difference between perceived current and desired knowledge levels (i.e., “the level of knowledge you desire” about medical cannabis). Rather than using averages, the knowledge gap was calculated based on how much greater an individual’s desired knowledge level was compared to their current knowledge level [36]. Only response pairs (i.e., current and desired knowledge) were used, and responses where the desired level was lower than the current level were excluded. To further elucidate, the knowledge gap was calculated by having each respondent’s current knowledge level response subtracted from their desired knowledge level response.

Prior to the onset of qualitative data analysis, the accuracy of the transcripts was checked by listening to the digital recordings. Content analysis was used to analyze the qualitative data [37], with two team members (AAA and LGB) independently reading the transcripts and developing a preliminary coding scheme. Constant comparison of new and existing data ensured consistency, relevance, and comprehensiveness of emerging codes. Several strategies were applied to ensure rigour in the qualitative analysis. To increase credibility, a team member with expertise in qualitative inquiry (LGB) monitored the qualitative data and its analysis. Confirmability was addressed by using the participants’ own words throughout the process of data analysis, interpretation, and description. An audit trail was kept documenting the activities of the study, including data analysis decisions.

## Results

### Environmental scan of cannabis-related policies

Administrators at the LTC facility provided the research team with the policies and procedures that addressed the management and use of medical and non-medical cannabis within the facility. The guiding policy adopted by the LTC facility was a generic policy applicable to all sites and facilities governed by a regional health authority. This policy, entitled “Patient Use of Medical Cannabis (Marijuana)” was issued in June 2020. The policy, which aimed to provide individuals with “reasonable access to medical cannabis”, outlined numerous issues that might arise with institutional cannabis use, including “ordering, labeling, packaging, storage, security, administration, documentation and monitoring requirements for the use of medical cannabis”. Key aspects of the policy are summarised in a table found in the Supplementary Material section (Supplementary Material 3).

Other relevant policies that were reviewed included the standards of practice issued by the provincial college of nurses and the college of physicians and surgeons [38–40], which provided direction to HCPs working in LTC about their scope of practice regarding medical and non-medical cannabis. The regional health authority’s smoke-free policy [41] also informed how inhaled forms of medical and non-medical cannabis were addressed, requiring residents to leave the facility grounds to smoke or vape cannabis. Lastly, the overarching federal Cannabis Act and Regulations provided guidance to both administrators and HCPs regarding the Canadian regulations specific to medical and non-medical cannabis [1, 42]. Together, existing facility, regional, and national policies created a context in which cannabis was framed as neither a medicine nor a controlled substance, but something unique and complex that must be navigated by residents, family members and staff in LTC settings.

### Quantitative survey

#### Demographic characteristics

From the approximately 318 eligible HCPs employed at the LTC facility, a total of 71 participants consented and completed the survey, yielding a response rate of 22.3%. With regards to response rate by profession, pharmacists (50.0%) and social workers (42.9%) were best represented, followed by physicians (23.1%), nurses (21.0%), and PT/OT (11.4%).

Most respondents were women (71.8%), registered nurses (62.0%) and worked within the palliative care unit (76.1%) at the facility. The average age of the sample was 40.9 years and the largest proportion of the sample had worked in the LTC facility for 5 or less years. See Table 1 for additional details.

**Table 1:** Participant demographic characteristics (*N* = 71)

Characteristics	Frequency (%)
**Gender**	
Woman	51 (71.8)
Man	17 (23.9)
Gender diverse	1 (1.4)
Do not wish to answer	2 (2.8)
**Profession**	
Registered Nurse	44 (62.0)
Physician	9 (12.7)
Licensed Practical Nurse	7 (9.9)
Social Worker	3 (4.2)
Occupational Therapist	2 (2.8)
Pharmacist	2 (2.8)
Physiotherapist	2 (2.8)
Other	2 (2.8)
**Practice area** ^**a**^	
Palliative care	54 (76.1)
Personal care home	20 (28.2)
Advanced dementia/behavioral unit (ACE)	18 (25.4)
Stroke rehabilitation	18 (25.4)
Chronic care	16 (22.5)
Acquired brain injury rehabilitation	15 (21.1)
Other	5 (7.0)
**Practice years at facility**	
0–5 years	30 (42.3)
6–10 years	12 (16.9)
11–15 years	14 (19.7)
16–20 years	9 (12.7)
>20 years	6 (8.5)
**Age**	
20–30	13 (18.3)
31–40	23 (32.4)
41–50	15 (21.1)
>50	14 (19.7)
Missing	6 (8.5)
Average age (SD)	40.9 (11.1)

^a^Participants could choose more than one option

#### Knowledge about medical cannabis

HCPs reported being most knowledgeable about the therapeutic potential of cannabis (3.1/5.0), potential risks of medical cannabis (2.9/5.0), and the different ways to administer medical cannabis (2.9/5.0). They reported being least knowledgeable about the dosing of medical cannabis (2.0/5.0), how to create effective treatment plans related to medical cannabis (2.1/5.0), and the similarities and differences between different forms of cannabis products and prescription cannabinoid medications (2.2/5.0). The top three ranked knowledge gaps mirrored the items ranked lowest with regards to knowledge (see Table 2). Overall, there was high interest in gaining more medical cannabis knowledge, with all knowledge items scoring greater than 4 on desired knowledge level.

**Table 2:** Medical cannabis knowledge scores and gaps

Knowledge items	Mean current knowledge score (*n* = 71)	Mean desired knowledge score (*n* = 58)	Mean knowledge gap^a^ (*n* = 58)
Dosing of medical cannabis for residents/patients	2.0	4.2	2.3
Creating effective treatment plans for residents/patients using medical cannabis	2.1	4.3	2.3
Similarities and differences between dried cannabis, other forms of cannabis products, and prescription cannabinoid medications	2.2	4.3	2.2
Mechanism of action of cannabis (endocannabinoid system)	2.2	4.3	2.2
Safety, warning signs and precautions for residents/patients using medical cannabis	2.5	4.4	2.0
Laws and regulations on use of medical cannabis	2.8	4.5	1.8
Potential risks of using cannabis for medical purposes	2.9	4.5	1.8^b^
Alternative routes of administration of medical cannabis	2.9	4.3	1.6^b^
Potential therapeutic use for cannabis	3.1	4.5	1.4

^a^Gap was calculated using individual response pairs = desired knowledge level minus current knowledge level. The means of the response differences are presented on this table

^b^*n*=57

#### Practice experiences with medical cannabis

About half of HCPs reported providing care in the past month to a resident who was taking medical cannabis (54.9%) and a quarter (25.4%) to a resident that was taking non-medical cannabis. Over 60% had been approached by a resident and/or a family member to discuss the potential use of medical cannabis; however, few HCPs reported initiating these conversations. Moreover, when asked if they felt comfortable discussing medical cannabis, 32.4% of HCPs disagreed (data not shown). Less than 20% reported helping residents, either directly or indirectly, to use medical cannabis and a very small proportion (1.3–2.8%) reported assisting residents’ consumption of non-medical cannabis. With regards to authorizing the use of medical cannabis or prescribing cannabinoid medication, which in Canada can be done by either a physician or nurse practitioner, just over half of physicians reported supporting residents’ access to these types of treatment. See Table 3 for additional details.

**Table 3:** HCPs’ practice experiences related to medical and non-medical cannabis (*N* = 71)

**Practice Experiences with Medical Cannabis**	**Yes** ***n*** **(%)**	**No** ***n*** **(%)**	**N/A** ^**a**^ ***n*** **(%)**
Approached by a resident/patient and/or their family to discuss the use of medical cannabis	44 (62.0)	26 (36.6)	1 (1.4)
Initiated a discussion with a resident/patient and/or their family on the use of cannabis for medical purposes	7 (9.9)	61 (85.9)	3 (4.2)
Provided care in the past 30 days to a resident/patient who is using medical cannabis	39 (54.9)	30 (42.3)	2 (2.8)
Indirectly assisted a resident/patient in the past 30 days to use medical cannabis (i.e., opened a bottle, read a label, provided water for swallowing pills, etc.)	14 (19.7)	51 (71.8)	6 (8.5)
Directly assisted a resident/patient in the past 30 days to use medical cannabis (i.e., administered cannabis product through an oral, rectal, or topical route)	13 (18.3)	50 (70.4)	8 (11.3)
Prescribed in the past a pharmaceutical cannabinoid medication to a resident/patient	5 (7.0)	4 (5.6)	62 (87.3)
• Nabilone (Cesamet®) • Nabiximols (Sativex®) • Dronabinol (Marinol®) • Cannabidiol (Epidiolex®)	5 (100.0) 2 (40.0) 2 (40.0) 0		
Supported in the past a resident’s/ patient’s access to Canada’s medical cannabis program (i.e., signed a medical declaration in support of an application for an authorization to possess cannabis for medical purpose).	2 (2.8)	7 (9.9)	62(87.3)
**Practice Experiences with Non-Medical Cannabis**	**Yes** ***n*** **(%)**	**No** ***n*** **(%)**	**N/A** ^**a**^ ***n*** **(%)**
Approached by a resident/patient and/or their family to discuss the use of non-medical cannabis	14 (19.7)	56 (78.9)	1 (1.4)
Initiated a discussion with a resident/patient and/or their family on the use of non-medical cannabis	6 (8.5)	62 (87.3)	3 (4.2)
Provided care in the past 30 days to a resident/patient who is using non-medical cannabis	18 (25.4)	51 (71.8)	2 (2.8)
Indirectly assisted a resident/patient in the past 30 days to use non-medical cannabis (i.e., opened a bottle, provided water for swallowing pills, provided an edible product, etc.)	2 (2.8)	67 (94.4)	2 (2.8)
Directly assisted a resident/patient in the past 30 days to use non-medical cannabis (i.e., administered cannabis product through an oral route)	1 (1.4)	65 (91.5)	5 (7.0)

^a^N/A was used to indicate practice behaviours not within an HCP’s scope of practice

#### Barriers to medical cannabis use in long-term care

Lack of knowledge, education or information about medical cannabis were reported to be barriers to medical cannabis use in LTC by most HCPs (81.7%). Moreover, the uncertain risks and benefits of medical cannabis and the lack of clinical guidelines were also perceived as barriers by 66.2% and 63.4% of HCPs, respectively. The complete list of barriers is presented in Table 4.

**Table 4:** Perceived barriers to the use of medical cannabis (*N* = 71)

Perceived Barriers	*n*^a^ (%)
Lack of personal knowledge/education or information regarding the use of medical cannabis	58 (81.7)
Risks and benefits are not sufficiently clear for potential therapeutic uses	47 (66.2)
Lack of clinical guidelines for the use of medical cannabis	45 (63.4)
Uncertainty about possible interactions with other medications	43 (60.6)
Insufficient information regarding the appropriate use of medical cannabis	41 (57.7)
Practice standards and/or policies from health professional associations or regulatory bodies	36 (50.7)
Uncertainty over whether cannabis has any therapeutic value	30 (42.3)
Belief that cannabis is not an appropriate treatment in a specific case	29 (40.8)
Potential liability concerns	29 (40.8)
Concern about possible side effects	29 (40.8)
Availability of prescription cannabinoids (e.g., Sativex®, Marinol® or Cesamet®)	20 (28.2)
Concern that patients who request medical cannabis may actually want it for recreational purposes	13 (18.3)
Other	4 (5.6)

^a^HCPs could select more than one response

#### Education about medical cannabis

Most of the HCPs agreed that additional education on medical cannabis would increase their comfort with discussing this treatment option with residents and family members (87.4%; data not shown). With regards to indirectly or directly administering medical cannabis to a resident, most HCPs for which this fell within their scope of practice also reported they would feel more comfortable if they had further education (59.2% and 56.4%, respectively; data not shown).

Over half of HCPs had not received any prior education related to medical cannabis (54.9%). Those that had, received it from conferences or workshops (65.6%), books or journal articles (43.8%) or through a colleague (37.5%). While almost half the sample (49.3%) reported receiving information from peer-reviewed sources, nearly a quarter received information about medical cannabis from a non-peer reviewed source or from a resident or family member. Some participants also received information from a cannabis industry source. Table 5 provides additional details.

**Table 5:** Previous education about medical cannabis (*N* = 71)

Items	Frequency (%)
**Previous education about medical cannabis**	
Yes	32 (45.1)
No	39 (54.9)
**Source of previous education about medical cannabis** ^**a,b**^	
Conferences/workshops	21 (65.6)
Books and journal articles	14 (43.8)
Colleagues	12 (37.5)
Undergraduate program	8 (25.0)
Graduate health professional training	4 (12.5)
Online training program	3 (9.4)
**Source of information on medical cannabis** ^**b**^	
Peer-reviewed sources	35 (49.3)
Non-peer-reviewed sources	15 (21.1)
Licensed producers	10 (14.1)
Community-based dispensaries	3 (4.2)
Patients and families	15 (21.1)
I do not access any information	16 (22.5)

^a^*n*=32; ^b^HCP could choose more than one response

**Fig. 1:**
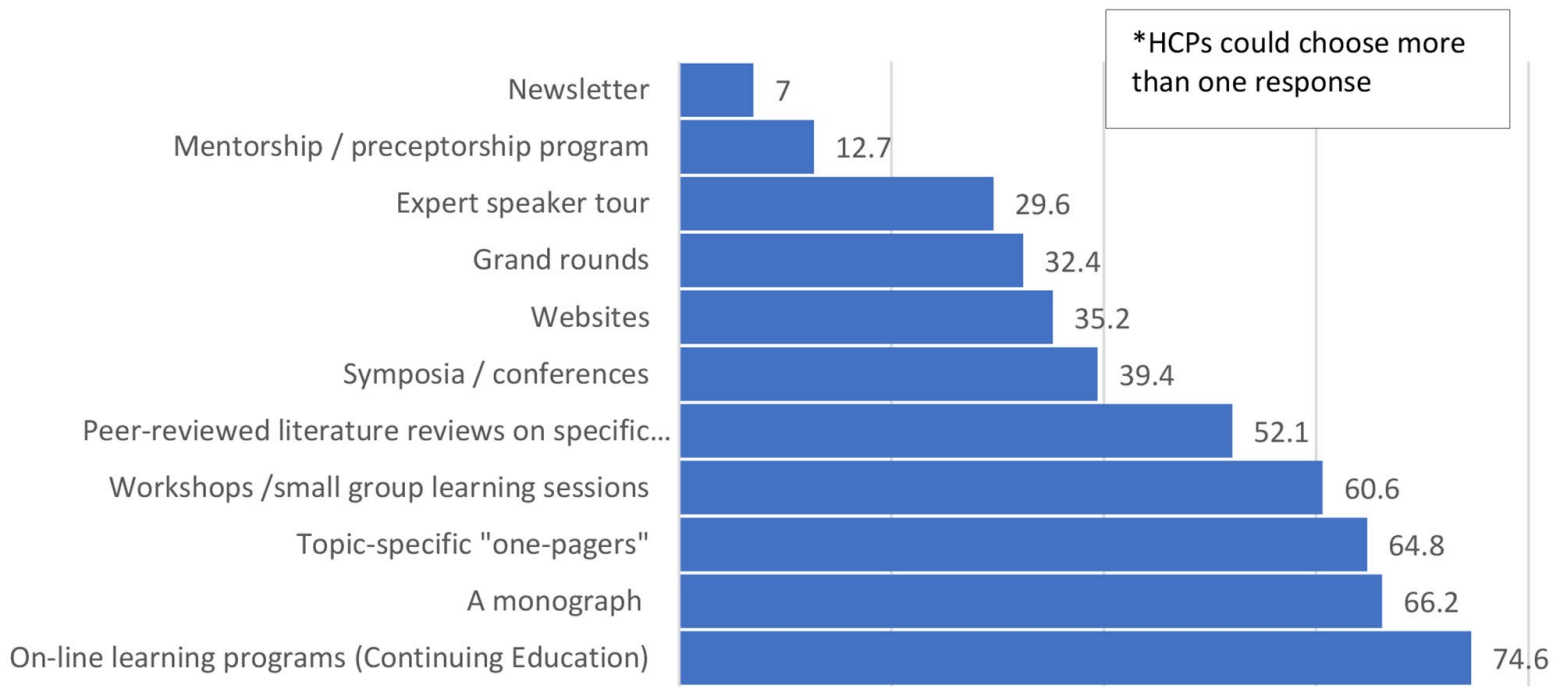
Percentage of respondents indicating prefered method of cannabis education*

The preferred sources of medical cannabis education were online learning programs (i.e., continuing education) (74.6%), monographs (66.2%), and topic-specific one-pagers (64.8%). See Fig. 1 for further details.

### Qualitative findings

A total of 12 HCPs were interviewed regarding their perceptions and experiences related to medical and non-medical cannabis in the LTC facility. This included 3 HCPs who were administrators, 6 nurses, 1 physician, 1 social worker and 1 pharmacist. Four main themes were identified.

#### Attitudes regarding medical cannabis: cautious support

There were mixed attitudes regarding the potential role of medical cannabis in general and in LTC populations. While some HCPs felt medical cannabis was a “good idea” for which there was beginning research regarding its health benefits, other HCPs believed additional high-quality evidence was needed prior to medical cannabis becoming a therapeutic option.

*I think it’s [medical cannabis] the fair option, it helps some people, but it doesn’t help others. So, I think we need a bit more evidence and a bit more research and having it available sort of allows for that research to occur* (Physician; PC07).

There appeared to be greater acceptance for medical cannabis use by individuals at end of life compared to those not considered immediately palliative (i.e., living with dementia, stroke, or traumatic brain injury), the latter of which comprise the majority of the people living in LTC settings. For individuals receiving palliative care, some HCPs perceived medical cannabis to be beneficial in managing pain, nausea, and anxiety, as well as reducing the use of other medications that may be problematic (e.g., opioids) due to their side effects. The potential value of medical cannabis in “adding quality of life and living” at the end of life was also mentioned.

*I’m working on the palliative care unit right now. A lot of patients that I’ve seen use it [medical cannabis] for anxiety purposes, or for nausea… some people find beneficial. So, I’ve seen it – people find it helpful for those reasons, and then they have to take less of their other medications. So, if it’s worked well for them and that’s what they prefer to do, then I think it should be an option for people, especially if some people find it beneficial.* (Registered nurse; PC03)

Within the context of LTC, several HCPs also spoke of the importance of respecting residents’ autonomy and previous experiences taking medical cannabis. The reality of a LTC facility being a resident’s “home” was particularly influential in HCPs’ support of medical cannabis being included as part of a holistic approach to care.

*I guess because people live at [LTC facility’s name], that is their home and if they were at home in the community, they would be able to access it [medical cannabis].* (Registered nurse, PC02)

*I think it’s a part of people’s lives. And I think if we’re allowing people to have certain things and keeping it as part of their treatment because if you look at a holistic view, preventing somebody from doing something that they’ve been doing for many years is not going to help them be accepting of other types of therapies.* (Pharmacist, PC09)

Some HCPs also perceived medical cannabis as offering an alternative to medical treatments that were not consistently effective in managing challenging health conditions, such as dementia and agitation.

HCPs’ attitudes towards medical cannabis varied across different products and routes of administration. Given the existing smoke-free policy at the facility, HCPs were more supportive of edibles, oils, oral sprays or topical creams and lotions than any form of inhaled medical cannabis (i.e., smoking and vaping). They were concerned not only about lung health, environmental exposure, and maintaining a scent-free facility, but also about how to safely manage vulnerable residents travelling off the facility’s property to smoke or vape.

#### Medical cannabis access and use: concern, confusion, and limited conversations

According to HCPs interviewed, most residents using medical cannabis obtained their authorization prior to moving to LTC. Individuals who sought authorization after arriving at the facility struggled to have their requests acknowledged or addressed by the health care team. As one nurse shared:

*I do remember I had a resident that did ask about it [medical cannabis]. And whenever it was kind of brought up, it didn’t seem to be acknowledged all the time. Or there were people who didn’t like the idea of having a resident on it.* (Registered nurse; PC06)

Conversations about medical cannabis were perceived to be severely limited by the culture surrounding medical cannabis at the LTC facility. The lack of open discussion about medical cannabis was seen by some to create conflict and negatively impact the development of trust between residents, family members, and the health care team: “*Without that discussion, it does create conflict within the team and between the physician and family, and perhaps that could impact the trusting relationship”* (Administrator; PA03). Further, several HCPs expressed the belief that conversations about non-pharmacological forms of medical cannabis could not be initiated by them due to policy issues; residents who expressed interest but did not have prior authorization were instead directed towards pharmaceutical forms of cannabis.

*There have been residents who have asked about using cannabis. And as I said, you can’t initiate it, if they’re going to get it on their own, fair enough. That’s pretty much been the experience I’ve had with residents with just non-pharmaceutical medical cannabis*. (Physician, PC07)

The only HCP-initiated conversations about medical cannabis mentioned were those occurring between pharmacists and residents, which focused on the potential side effects, benefits, and “red flags” to watch out for, such as allergic reactions.

HCPs shared that for those residents with authorization, they or a support person were responsible for ordering the medical cannabis product from an LP, which would then send the product to either the resident at the LTC facility or to their support person’s home. The cannabis product was then stored in a locked drawer in the resident’s room if they were self-administering or in a medication room if nursing staff were assisting with administration. According to one pharmacist, the pharmacy department was not permitted, due to existing federal regulations, to either directly order or dispense medical cannabis:

*No, we don’t dispense any cannabis. It’s considered resident’s own. So, we don’t acquire it for them. They have to directly be the holders of it and have it provided to them directly. And I think that has more to do with the regulations within Canada, the resident has to have certain type of documentation in order to have medical cannabis. So, it’s directly to them, we’re not able to order it for them or anything like that on their behalf.* (Pharmacist; PC09)

With regards to the type of medical cannabis products permitted in the facility, due to non-smoking policies and concerns about safety issues and the “smell”, combustible forms and inhaled routes of administration (i.e., joints, vaporizers, vape pens) were not allowed; instead, ingestible forms were mentioned most frequently by HCPs.

There was some confusion and concerns expressed regarding the storage and disposal of medical cannabis, which may have reflected changes in facility policies over time. Some HCPs expressed concerns about the storage of cannabis in residents’ rooms and the lack of “safeguards” to limit potential diversion and allow an accurate “count” of medical cannabis.

*We have to go into our Pyxis machine to retrieve a key to open that drawer. So, by going by that you’re able to know who’s actually accessed the key, but once the key is out you have no idea how many people have used that key and accessed that drawer before it’s gone back. You have no way of knowing how much cannabis has been taken out [of the drawer] or used, because you know there’s no way to measure it. So that’s a huge problem, I find.* (Registered nurse; PC01)

This nurse was particularly concerned about the potential risk of being accused of diversion:

*I’m not worried about people abusing it, it’s more the worry of being accused. You know, like, if a resident says, ‘why is my cannabis running out already, I thought I had enough for a few more weeks?’ and we’re like, ‘I don’t know’, right? There’s the potential for that sort of thing to happen.* (Registered nurse; PC01)

There was also a perception that there was a lack of direction from the facility regarding the appropriate disposal of medical cannabis. Most believed residents or family members were expected to remove any unused product once the resident was no longer at the facility. When such disposal was not possible, the policy was to destroy the cannabis product in a manner similar to narcotics or other controlled substances. However, variations in practice occurred with some HCPs described “throwing it in the trash” or using a medical waste disposal bin with or without a witness.

#### Barriers to medical cannabis use: safety, stigma and lack of knowledge

Numerous barriers to the use of medical cannabis by LTC residents were identified by HCPs. Foremost, the policies related to how cannabis products were ordered, accessed, stored, and administered were perceived to be complicated and created barriers to residents wanting to take medical cannabis, particularly those without family support. The inability of the LTC facility to order medical cannabis on behalf of a resident was perceived to be especially problematic, as described by one registered nurse:

*I know when it became legal, there were a few residents who have inquired about it, but they didn’t have the family resources in place to be able to get it because I believe there’s some hoops that you have to go through to be able to have it medically prescribed in getting it on to the unit. And so, the ones who were interested in it didn’t have those supports in place, so they weren’t able to get it prescribed for them.* (Registered nurse; PC05)

The lack of awareness and understanding of the regional policies related to medical cannabis by some of the clinical staff was also seen as being problematic. As one registered nurse shared:

*My only concern is that there’s a lot of rules around being able to administer and how it’s [medical cannabis] administered, which can again make things a bit complicated. I would say that’s probably my biggest concern is just it’s hard to remember everything that you have to do when you’re trying to administer it or helping a resident. So, you don’t get involved.* (Registered nurse; PC06)

Several HCPs attributed the lack of awareness about cannabis policy to the onset of the COVID-19 pandemic, which overshadowed all other health issues within their facility: “*Everybody’s been so focused on COVID for a year and a half that there hasn’t been really time to really think about or educate on other things.*” (Registered nurse; PC01).

HCPs suggested that more “straight forward” and tailored policies were needed that simplified how medical cannabis was managed. Having facility-specific policies would acknowledge the uniqueness of the LTC population, who may have cognitive impairment, limited social support, and complex healthcare plans. As one nurse shared: “*If it’s a dementia patient, they can’t really administer it on their own. So how do we follow the policy to help the patient take the cannabis? How would we know when they would want to take it PRN?”* (Registered nurse; PC03). It was also recommended that the policy that prevented the facility from directly ordering and supplying medical cannabis required revision so that LTC residents were not reliant on family members to gain access. Lastly, several HCPs suggested that medical cannabis policies need to be well advertised and additional training developed for clinical staff to enhance their awareness and comfort level in providing appropriate and supportive care.

*There needs to be a training session… staff have to read through them [cannabis policies] and get instructions about them, sort of like a self-learning activity. But that is not part of what we do when orienting.* (Registered nurse; PC02)

Another perceived barrier frequently mentioned by HCPs was their lack of knowledge regarding the potential risks and benefits of medical cannabis. There was limited understanding about the effects of medical cannabis, how it may interact with other medications and health conditions, what side effects could arise, as well as basic information about starting dose, titration, and difference between THC and cannabidiol (CBD). Without such information, HCPs were perceived to be very hesitant about recommending or supporting medical cannabis as a treatment alternative for LTC residents:

*There’s lots of unknown, that’s the problem. If there were more specifics about the recreational and the medical use of cannabis, then I think health care professionals would be more likely to want to provide it to the residents. But if not, then that’s kind of what’s hindering health care professionals to provide it.* (Registered nurse; PC08)

There was also substantial discussion by HCPs regarding the “stigma” that they perceived to exist within the facility regarding medical cannabis. As described by one pharmacist: “*I think the understanding of cannabis, regardless of if it’s medical or anything, it’s still considered in many people’s minds as an illicit drug. It hasn’t shaken that. And I think there’s a lot of stereotypes around the type of people that use cannabis”* (Pharmacist; PC09). The stigmatization of medical cannabis was perceived to be particularly pronounced among the medical staff, which led to what was described as a “hands-off approach” with regards to authorizing medical cannabis.

Almost all HCPs and administrators interviewed recommended that education programming and resources for HCPs be developed to address the lingering stigma associated with cannabis and the knowledge gaps that exist about medical cannabis and associated policies. Several participants recommended that education initiatives should first target physicians, who were responsible for authorizing medical cannabis in the facility. Physicians were perceived to need education on when and for whom medical cannabis would be appropriate, the latest evidence regarding efficacy and safety (i.e., drug interactions), and what their obligations and responsibilities were as the authorizing HCP. Participants also thought that all HCPs could benefit from additional training regarding medical cannabis, including the different types of cannabinoids and products, the process of titration, and dosing. Some of the nurses interviewed also expressed the need for education about the legal implications of medical cannabis and their role regarding provision and administration:

*I think the legal implications of cannabis use, I think that would be a good focus for the nursing group – so that they understood what their obligations were, what they could be held accountable for, those kinds of things.* (Administrator; PA02)

Finally, numerous HCPs spoke of the need for “safeguards” and clear policies and procedures to ensure that clinical staff were aware of what type of medical cannabis products residents were taking, what was the “right dose”, and the possibility of cannabis interacting with other medications. As shared by one pharmacist:

*So that we know that this patient is on it because there are potential drug interactions with other things that patients are taking. So, we just have to be cautious and aware that patients are doing this. Because especially right now with studies, there haven’t been a lot of great studies on drug interactions.* (Pharmacist; PC09)

#### Non-medical cannabis use: balancing autonomy and safety

HCPs were asked about their attitudes and experiences about residents’ use of non-medical cannabis in the facility. Two disparate points of view became apparent – those that perceived non-medical cannabis as a legal substance that should be available to LTC residents given the facility was their home and those that saw non-medical cannabis as a stigmatized substance that could lead to problematic use and disruptions in the care environment.

*Because it is somebody’s home and so you’re trying to honour and match what their lifestyle and aspects of their life at home were and matching that here [LTC facility]. The bad is, while it is somebody’s home, it’s the next person’s home too, and so it’s trying to balance that, right? In an institutional setting, trying to make it as home-like as possible but, at the same time, you know, monitoring and matching for what everyone’s needs are.* (Registered nurse; PA01)

*Professionally, I think that it creates issues in terms of trying to police the use of recreational cannabis. In terms of smoking cigarette tobacco, that’s an issue in itself. We’re a non-smoking facility. So, adding cannabis to the mix creates issues…having staff perhaps exposed or other people exposed if people are using cannabis indoors or where they’re not supposed. Or if they want to access and use cannabis outside, who’s going to take them for that? Because that creates exposure too for staff or others who may have to escort them.* (Registered nurse; PA03)

HCPs frequently mentioned the complexity of managing residents’ non-medical use of cannabis given the facility’s non-smoking policy that required residents to leave the facility grounds to use inhaled forms of cannabis. With staff unable to transport residents outside, concerns were raised regarding the safety of residents, particularly in the winter months, and who would be responsible for their transfer in and out of the facility as well as monitoring how much cannabis was consumed. In addition, residents’ access to non-medical cannabis was again dependent on having a support person that was able and willing to transport the product to the facility, posing a potential equity issue for some residents:

*If someone’s wanting to go smoke outside, then mobility might be an issue. If they don’t have the right wheelchair or family to take them outside for that. If they have the access. Like, if they need family to go and buy it and bring it to them, that could be more of an access issue depending on their family support.* (Registered nurse; PC03)

There was specific concern expressed for individuals in the rehabilitation units who may have pre-existing substance use issues. For these individuals, HCPs were concerned that allowing access to non-medical cannabis could add to an already complex care plan. In addition, with many vulnerable residents living at the facility, concerns were raised regarding them being “incredibly suggestible” to others encouraging their consumption of cannabis:

*These people – they have an addiction. For sure they’re making choices, but those choices are influenced by physical withdrawal or influenced by stress; they’re influenced by lots of things. So, I would hate to put residents in a position where that was one other [non-medical cannabis] thing they had to contend with during the rehab stay.* (Administrator, PA02)

## Discussion

The use of cannabis for therapeutic and recreational purposes is becoming more prevalent within older adult populations, both in the community as well as within healthcare institutions. There has also been growing interest in the possible role of medical cannabis for select chronic, rehabilitative, and palliative health conditions, frequently found among individuals residing within LTC settings. LTC facilities, thus, face the complex practice and policy implications associated with a substance that has been surrounded in controversy for close to a century. This case study is among the first to explore in one LTC facility in Western Canada how cannabis use is being addressed following the legalization of non-medical cannabis products, and what challenges exist. It provides an important snapshot of the complexities surrounding cannabis use in LTC and a foundation for future research.

### Cannabis use in LTC settings: a clash of cultures

One challenge experienced by people residing in LTC facilities is the tension that exists between social and medical models of care that most facilities are founded on. Historically, LTC facilities have operated as what Goffman [43] termed “total institutions”, places where every aspect of a person’s life was controlled by others, paternalism dominated, and the medical needs of people were what drove care practices. Aspects of the total institution still exist, as noted in this case study, whereby cannabis use is in the control of the HCPs; it is dispensed during medication administration times rather than being freely available for use by the resident when they so desire as would be in a person’s home. In trying to create more home-like environments and meet the broad range of social and emotional needs of residents, resident-centred care practices and relational models of care have emerged [44]. Within this milieu, resident autonomy and choice are at the forefront and HCPs are there to assist, rather than take control of residents’ daily lives. In the most ideal settings, behaviours that are considered ‘risky’, like alcohol consumption, are treated as social experiences, not care tasks to be managed [45]. The tension arises, however, that despite the desire to be resident-centred, most LTC facilities are highly regulated by governments, putting limits to resident choice and, therefore, their autonomy [45]. While HCPs in our study acknowledged that residents should have the right to use medical or non-medical cannabis, the regional and institutional policies surrounding safety and the rights of other residents and staff to not be exposed to potentially risky behaviour underscored many of their views. LTC facilities would be wise to consider the principles of dignity of risk [46] with relation to cannabis consumption/use along the frail elderly population that reside in the home.

### Cannabis policies and LTC: one size doesn’t fit all

The cannabis policies developed at the advent of legalization, without consideration of the unique populations and healthcare challenges that exist within LTC facilities, created numerous barriers to residents accessing and using cannabis, as well as for HCPs attempting to provide appropriate care. One of the most significant challenges experienced by LTC residents in our study was the inability to obtain a medical cannabis authorization from a physician working in the facility. Another significant challenge was the regional policy that medical cannabis could not be couriered directly to the LTC pharmacy; instead, the resident or their support persons were responsible for ordering and bringing cannabis products into the facility. Both challenges created enormous inequity in which residents that lacked the physical and cognitive ability to obtain authorization and order medical cannabis from an LP or were without a support person willing and able to obtain medical cannabis on their behalf, were unable to access medical cannabis. Given the nature of LTC populations, these policies led to only a few residents being able to access and use medical cannabis as part of their care.

Another policy that had substantial safety implications for residents wanting to use inhaled forms of cannabis was the regional and institutional no smoking policies that prevented both tobacco and cannabis products from being consumed within the centre as well as on the grounds. As a result, residents had to make their own way, or be accompanied by a support person, to walk approximately 300 m to the public sidewalk where they were allowed to smoke or vape cannabis. With the LTC facility located in a region where winter temperatures can reach − 35 Celsius and sidewalks are covered in snow and ice, this poses significant risk for residents who may be at heightened risk of falls and utilizing assisted walking devices. Similar safety implications of smoke-free policies have been identified in previous research [47].

Lastly, the policies surrounding the storage and self-administration of medical cannabis for those residents with the physical and emotional capacity (or with a support person willing to administer) may pose potential safety and liability risks and contribute to the concerns held by some HCPs about the use of cannabis in LTC. While residents’ autonomy must be respected, as well as their own expertise with regards to medical cannabis use, the value of standardized medication protocols to ensure the safety of residents as well as to inform care decisions must be acknowledged. The tension experienced in balancing LTC residents’ autonomy with health and safety concerns in the context of substance use has been cited in a recent scoping review [48] as well as prior research that has examined the use of tobacco in residential care settings [49].

The policy-related challenges identified by study participants suggest that consultations with LTC residents, families and HCPs are urgently needed to develop and refine cannabis policies that address the needs and reality of individuals living and receiving care in LTC. Future policy reviews must balance LTC residents’ autonomy with the safety issues associated with cannabis use (i.e., dignity of risk), particularly among older adults and those with cognitive and physical impairments. Approaching cannabis policies and procedures in LTC from a harm reduction perspective [50] with regards to supporting safer consumption of medical cannabis (e.g., route of administration, designated consumption areas) may also be important. Further, the unique context of LTC must also be acknowledged in that for many residents, a LTC facility is their home, and will continue to be so until the end of their lives. But the shared nature of a LTC setting requires that some boundaries be established to protect all residents, as well as those working within LTC. From a staff perspective, a review of policies related to the administration and documentation of cannabis use is needed to protect them from claims of diversion as well as other medicolegal challenges.

### Cannabis knowledge gap and stigma in LTC

Across both the quantitative and qualitative data, the gap in knowledge regarding cannabis and the need for continuing education for HCPs working in LTC were readily apparent. When HCPs are unfamiliar about the various forms of medical cannabis, appropriate dosing and titration schedules, and routes of administration, they are hindered in their ability to engage in shared decision making with LTC residents as well as provide high-quality care [51–54]. Education is particularly needed that is tailored to the unique risks and benefits of medical cannabis use among LTC populations, including those living with physical and cognitive impairment. Older adults may be more sensitive to the side effects of cannabis due to changes in how medications and drugs are metabolised, and the predominance of polypharmacy among those residing in LTC may further complicate how individuals respond to cannabis [55]. Therefore, HCPs working in LTC must be aware of how cannabis use may impact individuals’ mobility, memory, and behaviour, as well as the potential for dependency, particularly among those who have experienced substance use issues in the past.

Beyond basic education regarding cannabis and its effects, HCPs must also become aware and informed about existing federal, regional, and institutional policies as well as professional practice standards regarding both medical and non-medical cannabis. The study findings highlighted the uncertainty many HCPs experienced regarding how medical and non-medical cannabis was to be accessed, authorized, administered, stored, and disposed within the LTC facility and what was within their professional scope of practice. Legal concerns about liability, workplace safety, and diversion were also raised.

It is important that future cannabis education programs targeting LTC settings also address the underlying stigma and stereotypes that still surround cannabis use [56, 57], despite the existence of a medical cannabis program in Canada for over 20 years and the recent legalization of non-medical cannabis. Experiential training that promotes non-judgmental communication that avoids stigmatizing language (e.g., user, addict, marijuana) and considers both the risks and benefits of cannabis use, particularly within the context of end-of-life care, will help address the stigma that HCPs and LTC residents and families may hold towards cannabis.

With the legalization of cannabis in many regions around the world, it is imperative that undergraduate health professional training programs include information about both medical and non-medical cannabis. Currently, there is a knowledge gap among HCPs due to the lack of standardized curriculum for medical cannabis across nursing or medical schools [35, 58]. Understanding such foundational knowledge such as the endocannabinoid system, the different forms and types of cannabis, and the potential health effects will enable physicians, nurses, pharmacists and other HCPs to engage in informed conversations with individuals and families both within and beyond LTC [33]. In addition, the development of continuing education programs focused on cannabis will ensure practicing HCPs have current knowledge about cannabis, including existing policies and programs relevant to medical and non-medical cannabis. For example, the Canadian Coalition for Seniors’ Mental Health created asynchronous e-learning modules to provide evidence-based knowledge for various clinicians [59].

### Non-medical cannabis use in LTC: it’s legal but…

Despite non-medical cannabis being a legal substance for over three years in Canada at the time of the case study, the use of non-medical cannabis by LTC residents was considered controversial amongst the HCPs interviewed. Not only were HCPs limited in their ability to support the use of non-medical cannabis due to regional policies that prohibited non-medical cannabis consumption at any healthcare facility and surrounding grounds but concerns about potential safety risks and disruptions to the care environment made some HCPs hesitant about supporting residents’ use of non-medical cannabis.

Notwithstanding these challenges, at least a quarter of HCPs surveyed reported providing care to a LTC resident who used non-medical cannabis, which suggests that regulatory and policy changes are required to ensure there is equity across LTC residents who may express interest in non-medical cannabis, as well as to address the unique safety and care issues associated with recreational cannabis use in LTC populations. Similar to medical cannabis, LTC residents’ autonomy must be considered in future policy changes related to non-medical cannabis to facilitate care that is free from stigma and bias, respects residents’ rights to make informed decisions and to live with risk, and to create a home-like environment where residents can engage in activities that were an important part of their lives before entering LTC.

Lessons can be drawn from literature that has examined the use of other legal substances, such as alcohol and tobacco in LTC [48, 60], and the need to develop person-centered care plans that ensure the safety of the individual, fellow residents, and the healthcare team.

## Limitations

Like all case studies, the findings cannot be extrapolated to other LTC settings and populations. Given that this study was undertaken in Canada, which has a socialized healthcare system and legalized both medical and non-medical cannabis, the experiences and attitudes of HCPs who participated may be unique and limit the generalizability of the findings. However, there are lessons to be learned regarding the challenges that residents in LTC facilities face in using medical and non-medical cannabis, as well as the potential need for both education and policy reform to better support HCPs in providing appropriate, safe, and person-centred care of LTC residents. In addition, the collection of both quantitative and qualitative data allowed triangulation during the data analysis and helped improved the rigor of the findings [61]. Recruitment and data collection for this study also occurred during the height of the COVID-19 pandemic. Therefore, the response rate was lower than desired and there was limited diversity among study participants with regards health profession designation. However, the proportion of physicians, nurses, pharmacists, and other allied health professions reflected the overall staff composition of the LTC facility.

## Implications for future research

Beyond the policy and practice implications discussed earlier, the study findings also point to the urgent need for research focused on cannabis use among populations commonly found within LTC settings. The lack of evidence regarding the potential health effects of cannabis in the management of diseases such as dementia, arthritis, Parkinson’s, traumatic brain injury, and multiple sclerosis led many of the HCPs interviewed to be hesitant about authorizing and supervising cannabis use for LTC residents living with these conditions. While there is a growing number of studies being undertaken focused on medical cannabis, many are limited by their sample size and study design. It is only through high-quality clinical trials that evaluate the efficacy and safety of medical cannabis that a change in practice will occur.

Future medical cannabis research must also be developed in a manner that is inclusive of older adults and those living in LTC. The exclusion of such populations from clinical research has been previously identified as problematic [62], resulting in research findings that lack generalizability and pose challenges in determining the applicability of research to older adults who may be living with numerous co-morbidities and using multiple medications. While the inclusion of older adults in medical cannabis clinical trials may be more methodologically and ethically challenging, it will lead to evidence that will inform both future policies and practices.

Lastly, our case study offers insight into the reality and challenges of cannabis use by residents of one LTC facility. Additional research across different jurisdictions is needed to explore how LTC settings are addressing cannabis use and to learn from their experiences. We encourage the continued use of mixed methods study designs to ensure the experiences and perspectives of residents, family members and HCPs are captured alongside administrative data related to medical and non-medical cannabis use.

## Conclusion

With the legalization of medical and non-medical cannabis in jurisdictions around the world, LTC facilities will be obligated to develop policies, procedures and healthcare services that are able to accommodate residents’ use of cannabis in a respectful and evidence-informed manner. Balancing the safety concerns against the potential therapeutic value of cannabis, as well as considering residents’ autonomy and the home-like environment of LTC, will be important considerations in how cannabis use is addressed and regulated. Our case study highlights the lack of knowledge, inequities, and stigma that continue to surround cannabis in LTC. There is an urgent need for research that not only explores the potential risks and benefits of cannabis, but also informs the development of more nuanced and equitable policies and education resources that will support reasonable and informed access to medical and non-medical cannabis for older adults and others living in LTC.

### Electronic supplementary material

Below is the link to the electronic supplementary material.

Supplementary Material 1

## Data Availability

The datasets generated and analysed during the current study are not publicly available due to the small sample size drawn from one health care facility but are available from the corresponding author on reasonable request.

## Abbreviations

CBD: Cannabidiol
HCP: Healthcare provider
LTC: Long–term care
THC: Tetrahydrocannabinol
LP: Licensed Producer
