# Supplementary material for: Cannabis use in a Canadian long-term care facility: a case study
Source: BMC Geriatr. 2024 May 29;24:467. doi: 10.1186/s12877-024-05074-2 (PMC11134741; doi:10.1186/s12877-024-05074-2)
Supplement: Supplementary file 1 — Supplementary Material 1 [file 12877_2024_5074_MOESM1_ESM.docx]

**Supplementary Material 1**

**Health Care Professional Survey**

Knowledge about the medical use of cannabis

1. Please circle the number that you feel best indicates your current level of knowledge and the level of knowledge you desire for the following topics: (1=very poor, 2=poor, 3=fair, 4= good, 5=very good)


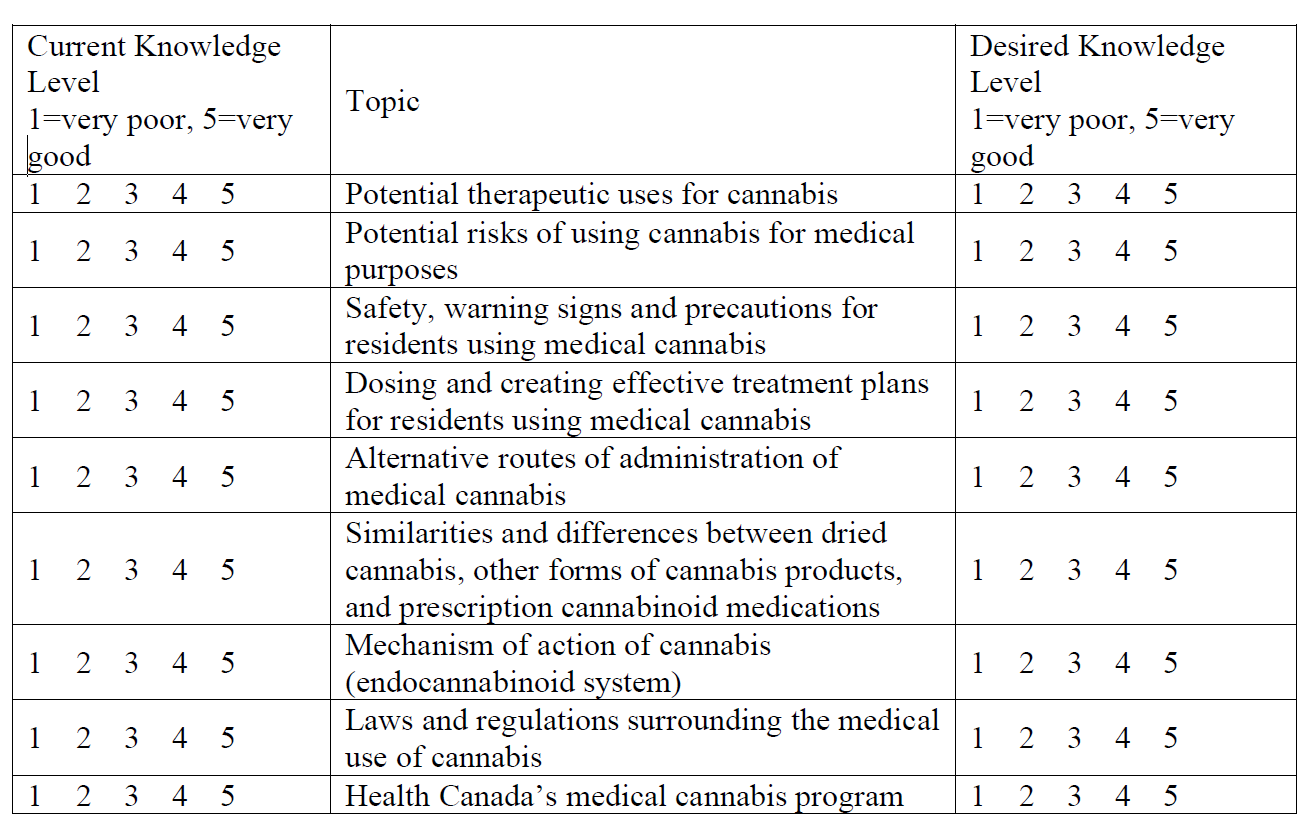


2. How strongly do you feel you need education on cannabis in health care?

Not at all

Not very strongly

Neutral

Strongly

Very strongly

**Experiences**

3. Have you ever been approached by a resident and/or his/her family to discuss the use of cannabis for medical purposes?

Yes

No

4. Have you ever initiated a discussion with a resident and/or his/her family on the use of cannabis for medical purposes?

Yes

No

5. Do you have resident using cannabis for medical purposes?

Yes

No

6. In the past, if you were able to, would you have ever prescribed a pharmaceutical cannabinoid

medication?

Yes

No

7. If you were able to, would you feel comfortable prescribing a pharmaceutical cannabinoid medication? (Check all that apply)

Nabilone (Cesamet®)

Nabiximols (Sativex®)

Cannabidiol (Epidiolex®)

Would not prescribe a cannabinoid

8. In the past, if you were able to, would you have ever supported a patient's access to Health Canada's medical cannabis program, that is, signed a medical declaration in support of an application for an authorization to possess cannabis for medical purposes?

Yes

No

9. If you were able to, would you feel comfortable supporting a patient's access to Health Canada's medical cannabis, that is, sign a medical declaration in support of an application for an authorization to possess cannabis for medical purposes?

Yes

No

**Barriers**

10. What barriers do you face regarding the use of medical cannabis? Please select all that apply.

􀀀 Risks and benefits are not sufficiently clear for potential therapeutic uses

􀀀 Potential liability concerns

􀀀 Lack of personal knowledge/education or information regarding the use of cannabis for medical purposes

􀀀 Instruction from nurse practitioner, nursing or medical associations or licensing bodies

􀀀 Belief that cannabis is not an appropriate treatment in a specific case

􀀀 Lack of clinical guidelines for the use of cannabis for medical purposes

􀀀 Availability of prescription cannabinoids (e.g. Sativex®, Marinol® or Cesamet®)

􀀀 Uncertainty over whether cannabis has any medicinal value

􀀀 Uncertainty about possible interactions with other medications

􀀀 Concern about possible side effects

􀀀 Concern that residents who request medical cannabis may want it for recreational use

􀀀 Insufficient information regarding the appropriate use of cannabis for medical purposes

􀀀 Not part of my scope of practice

􀀀 Other, please specify: ______________

**Attitudes**

11. Please indicate whether you believe the following healthcare professionals should be authorized to approve the medical use of cannabis:

| **Type of health care professional** | **Yes** | **No** |
| --- | --- | --- |
| Primary Care Physicians/Family Physicians |  |  |
| Specialist Physicians |  |  |
| Nurse Practitioners |  |  |
| Naturopathic Doctors |  |  |
| Registered Nurses |  |  |
| Pharmacists |  |  |

12. Please indicate any other health care professionals you feel should be authorized to approve the medical use of cannabis: _______________________________

13. **Physicians/Nurse Practitioners ONLY.** Please indicate the degree to which you agree or disagree with these statements: (1=Strongly agree, 2=Agree, 3=Neutral, 4=Disagree, 5=Strongly disagree)

| **Statement** | Strongly Strongly  agree disagree | | | | |
| --- | --- | --- | --- | --- | --- |
| I would feel more comfortable discussing the medical use of cannabis with residents / family members if I had more education about it | 1 | 2 | 3 | 4 | 5 |
| I would feel more comfortable about recommending medical cannabis if Health Canada offered me protection from liability. | 1 | 2 | 3 | 4 | 5 |
| I would feel more comfortable if physicians/nurse practitioners who participated in the medical cannabis were required to undergo a specific training or licensing program. | 1 | 2 | 3 | 4 | 5 |
| I feel that with more education I would be better able to treat patients using medical cannabis. | 1 | 2 | 3 | 4 | 5 |

**Educational approaches**

14. Please indicate the format(s)/structure(s) you would prefer for receiving further information. Select all that apply.

􀀀 A monograph on cannabis (similar to a drug product monograph)

􀀀 Topic-specific "one-pagers"

􀀀 Newsletter

􀀀 Peer-reviewed literature reviews on specific topics

􀀀 On-line resources

􀀀 On-line learning programs as part of Continuing Education (CE)

􀀀 Workshops/small-group learning sessions

􀀀 Symposia/conferences

􀀀 Expert speaker tour

􀀀 Grand rounds

􀀀 Mentorship / preceptorship program

􀀀 Other, please specify: _______________________________________

**Demographic Information**

15. What is your professional designation?

Physician (MD)

Nurse Practitioner (NP)

Social Worker

Occupational Therapist

Other (please specify): _______________________________________

16. What is your primary area of practice (check all that apply)?

Personal Care Home

Dementia

Stroke Rehabilitation

Acquired Brain Injury Rehabilitation

Palliative Care

Chronic Care

Other, please specify: ___________________________

17. How many years have you practiced as a health professional?

0-5 years

6-10 years

11-15 years

16-20 years

21 years or more

18. How many years have you practiced at [name of facility]?

0-5 years

6-10 years

11-15 years

16-20 years

21 years or more

Additional comments or opinions you would like to express:

______________________________________________________________________________

**Thank you for taking the time to complete this survey.**

**Supplementary Material 2**

**Interview/Focus Group Guide – Health Care Professionals**

1. What first comes to mind when I say “cannabis/marijuana/pot/etc.”?

2. What are your thoughts about cannabis being available as a medical therapy in Canada?

3. What are your thoughts about the legalization of cannabis for recreational use in Canada?

4. Do you think that medical cannabis should be available at a therapy option at [LTC facility name]? Why/why not?

5. What type(s) of medical cannabis, if any, do you think should be available at [LTC facility name]? and why?

a. Dried flower/smoking

b. Dried flower/vaping

c. Oil/gel caps

d. Oral sprays

e. Other

6. What has been your experience related to medical cannabis at [LTC facility name]??

a. Residents/family members’ asking questions about medical cannabis

b. Residents/family members’ requesting access to medical cannabis

c. Residents using medical cannabis

d. Authorizing medical cannabis

e. Providing medical cannabis (direct/indirect)

f. Follow-up care related to medical cannabis

g. Disposal/storage

7. What do you see to be the barriers to medical cannabis use at [LTC facility name]?? What would potentially support the use of medical cannabis at [LTC facility name]??

a. Policies

b. Health care professionals

c. Stigma

d. Risks/benefits

e. Information/evidence

f. Structural issues, like smoking areas, storage, etc.

8. What questions/educational needs do you have about medical cannabis use?

9. Do you think that recreational cannabis used by residents should be permitted at [LTC facility name]?? Why/why not?

10. What type(s) of recreational cannabis, if any, do you think should be permitted to be used by residents at [LTC facility name]?

a. Dried flower/smoking

b. Dried flower/vaping

c. Vaping products

d. Oil/gel caps

e. Oral sprays

f. Edibles

g. Other

11. What has been your experience related to recreational cannabis use at [LTC facility name]??

a. Residents/family members asking questions about recreational cannabis use

b. Residents/family members requesting use of recreational cannabis

c. Residents using recreational cannabis use

d. Storing/disposal recreational cannabis product

12. What do you see to be the barriers to recreational cannabis use at [LTC facility name]?? What would support the use of recreational cannabis at [LTC facility name]??

a. Policies

b. Health care professionals

c. Stigma

d. Risks/benefits

e. Lack of information/evidence

f. Structural issues, like smoking areas, storage, etc.

13. What questions/educational needs do you have about recreational cannabis use?

14. Do you have anything else to share related to either medical or recreational cannabis use by [LTC facility name]? residents?

**Supplementary Material 3**

**Table. Summary of Medical and Non-Medical Cannabis Institutional Policy**

| **Policy and Practice Standards**  **(Date)** | **Key Themes** | | | | | |
| --- | --- | --- | --- | --- | --- | --- |
|  | **Authorization of Medical Cannabis** | **Use of Medical and Non-Medical Cannabis** | **Access to Cannabis Products** | **Documentation of Cannabis Use** | **Storage of Cannabis Products** | **Disposal of Cannabis Products** |
| Regional Health Authority Policy on Medical Cannabis  Patient use of Medical Cannabis (Marijuana)  (June 2020) | Physicians and NPs considering authorization are directed to their colleges’ standards of practice related to medical cannabis.  HCPs shall assess the safety of medical cannabis given resident’s medical condition and other current therapies. | Medical cannabis use requires medical authorization document.  Permitted under the following conditions:   - Order from a prescriber documented in health record. - Authorization document documented in health record. - Secure location to store medical cannabis in care area.   Smoking and vaping prohibited, but exemptions possible.  Residents encouraged to self-administer or have a support person assist with administration. Nurses may administer when resident is unable to do so, and a support person is not available.  Shall occur only in a resident’s room or other authorized location.  Non-medical cannabis use is prohibited in regional health care institutions. | Purchased and provided by resident or support person.  Shipments from an LP are not allowed, except to LTC facilities.  Only product from a LP in original labelled container permitted.  Cannot be shared with other residents, staff, or visitors. | Medical cannabis use should be documented at admission as part of best possible medication history.  Orders are required to be documented in the health record with regards to:   - Self-administration or with assistance from a support person - Nurse administration (formulation, dose, route, frequency, and indication)   Verification of authorization form and registration document from LP is required once per admission or when a new or updated document is provided. | Medical cannabis that is self-administered by resident or with assistance from a support person shall be securely stored in a locked storage unit in a resident’s room. If not available, it will be stored in medication room.  Self-administered medical cannabis (with assistance of support person) will be documented as “patient’s own medication” on medication administration record and nurses shall confirm and sign once per shift that self-administration is on-going.  Medical cannabis administered solely by nurse will be stored in the medication room.  Presence of medical cannabis in the medication room will be recorded and double signed by nurses, whenever counts are performed on a narcotics/controlled substance form. Actual quantity of medical cannabis does not need to be recorded. | Upon discontinuation or discharge, medical cannabis will be returned to resident or support person, who may also request disposal, which will be done as per site procedures for disposal and documentation of narcotics and controlled substances.  In the event of a resident’s death, medical cannabis shall be discarded and documented. |
